# Distinct Transcriptional Programs Controlled by *NR5A1* and β-Catenin in Adrenocortical Carcinoma

**DOI:** 10.3390/medsci14040493

**Published:** 2026-08-19

**Authors:** João Carlos Degraf Muzzi, Bonald Cavalcante Figueiredo, Jean Silva de Souza Resende, Igor Samesima Giner, Mauro Antônio Alves Castro, Enzo Lalli

**Affiliations:** 1 Oncology Division, Instituto de Pesquisa e Faculdades Pelé Pequeno Príncipe, Curitiba 80250-060, PR, Brazil; bonaldf@yahoo.com.br (B.C.F.); jean.s.s.resende@gmail.com (J.S.d.S.R.); igor.sginer@gmail.com (I.S.G.); 2 Bioinformatics and Systems Biology Laboratory, Federal University of Paraná, Curitiba 81531-980, PR, Brazil; 3 Institut de Pharmacologie Moléculaire et Cellulaire CNRS UMR7275, Inserm U1323, Université Côte d’Azur, 06560 Valbonne, France

**Keywords:** adrenocortical carcinoma, *NR5A1*, SF-1, β-catenin, *LEF1*, *TCF7*, transcriptional regulatory network

## Abstract

Background/Objectives: Adrenocortical carcinoma (ACC) is a rare malignancy in which overexpression of steroidogenic factor-1 (SF-1/*NR5A1*) and aberrant activation of canonical Wnt/β-catenin signaling are important oncogenic events. However, the extent to which these regulatory axes converge or interact at the transcriptional level remains unclear. We investigated their relationship using bulk transcriptomic and regulatory-network approaches. Methods: Regulatory network inference using RTN/ARACNe was applied to the TCGA-ACC cohort. *NR5A1* and β-catenin-associated TCF/LEF regulon activities were evaluated in perturbation datasets and tested for associations with CpG island methylator phenotype (CIMP), overall survival, and gene-level interaction effects. Candidate modulators of *NR5A1* activity were assessed using the MINDy algorithm. The effects of cBAF inhibition on *NR5A1* regulon activity were also evaluated in H295R and CU-ACC1 cells. Results: *NR5A1* knockdown repressed steroidogenic pathways, whereas β-catenin knockdown predominantly suppressed Wnt/β-catenin signaling. In TCGA-ACC, *NR5A1* regulon activity was associated with CIMP-high status and overall survival. In contrast, β-catenin-related regulons were not significantly associated with CIMP-high status after adjustment for *NR5A1* activity, and no significant multiplicative interaction effects were identified. Fewer than 3% of protein-coding genes showed improved fit in models including *NR5A1* × TCF/LEF interaction terms, without enrichment for steroidogenic or Wnt/β-catenin-related pathways. *CTNNB1* was identified as a statistically significant but low-ranking positive modulator of *NR5A1* activity, whereas *CTNNBIP1* was a top-decile negative modulator. cBAF inhibition was associated with *NR5A1* regulon repression in both cell models. Conclusions: These findings indicate limited detectable global transcriptional convergence between *NR5A1* and canonical β-catenin-related regulons in the evaluated bulk transcriptomic frameworks. Potential relationships between these pathways may depend on more localized, chromatin-dependent, protein-level, or context-specific regulatory mechanisms. *NR5A1* regulon activity showed more consistent associations with CIMP-high status and clinical outcome than the β-catenin/TCF-LEF regulons in the evaluated TCGA-ACC models. The modulation patterns associated with *CTNNB1* and *CTNNBIP1*, together with the repression of NR5A1 regulon activity following cBAF inhibition, raise the possibility that NR5A1 acts as a context-dependent regulatory node connecting oncogenic signaling and chromatin-remodeling mechanisms in ACC. These findings support further investigation into the role of chromatin-remodeling complexes in sustaining NR5A1-driven transcriptional programs in ACC.

## 1. Introduction

Adrenocortical carcinoma (ACC) is a rare endocrine malignancy. Genomic studies have allowed to identify several factors which have an important role in the pathogenesis and malignant spread of ACC. Two key drivers have emerged from this body of work: overexpression of the transcription factor Steroidogenic Factor-1 (SF-1)/*NR5A1* and aberrant activation of the canonical Wnt signalling pathway [1].

Elevated *NR5A1* levels are a hallmark of paediatric ACC, while they are associated to a malignant clinical evolution in adult patients with ACC [2]. Remarkably, raising *NR5A1* expression above normal thresholds in ACC cells is sufficient to drive a transcriptional programme associated with aggressive tumour behaviour [3,4].

Conversely, constitutive activation of the canonical Wnt pathway, caused by somatic mutations in *CTNNB1* or genes encoding other pathway components, is detected in about one third of ACC cases, isolated or co-occurring with additional genomic alterations [5,6]. Aberrant β-catenin activity is associated to clinical significance in ACC, as it is significantly correlated with unfavourable patient outcomes [7]. Mechanistically, activated β-catenin enters the nucleus and co-operates primarily with TCF/LEF family transcription factors to drive downstream gene expression, though interactions with other classes of transcription factors, including nuclear receptors, have also been documented [8,9]. Importantly, *NR5A1* and β-catenin target genes have been identified in the H295R ACC cell line by knockdown experiments [4,10,11].

The concept of interaction in gene regulation is inherently multifaceted and context-dependent. Interactions may occur at different biological levels, including chromatin occupancy, protein–protein interactions and transcriptional regulation and may reflect either direct physical association or functional dependency between regulatory elements.

From a statistical perspective, interaction can be evaluated through different frameworks, including additive or multiplicative effects between variables. However, the complexity of biological systems often precludes straightforward conclusions regarding dependence or independence between regulatory programs.

This conceptual complexity is reflected in the current literature on *NR5A1* and β-catenin in ACC. While some authors [12,13] proposed a cooperative model supported by chromatin-level and transcriptional evidence, Lalli suggested that these factors regulate largely distinct gene sets, supporting a model of functional independence of *NR5A1* vis-à-vis β-catenin [14].

In this study, we evaluated the relationship between *NR5A1* and β-catenin in ACC at the level of transcriptional output, with the goal of investigating whether these regulatory axes show evidence of transcriptional convergence or statistical interaction in bulk transcriptomic data. Given this unresolved debate, we considered two non-mutually-exclusive working hypotheses: (i) that NR5A1 and β-catenin/TCF-LEF regulons converge on a shared transcriptional programme detectable in bulk tumour data, consistent with a cooperative model, or (ii) that broad transcriptional convergence between these regulatory axes is not readily detectable at the bulk level, with potential relationships confined to chromatin-, protein-, or context-dependent mechanisms not captured by broad multiplicative interaction terms.

To distinguish between these possibilities, we applied complementary computational approaches, including regulatory network inference, perturbation-based regulon activity analysis, pathway enrichment, gene-level interaction models, modulator inference and association analyses with CIMP status and overall survival. This strategy was designed to evaluate potential relationships between these axes across transcriptional, phenotypic and clinical dimensions.

## 2. Materials and Methods

### 2.1. Data Acquisition for Differential Expression Analyses of Perturbation Models

For the *NR5A1* knockdown experiment in H295R cell line, phenotypic and expression data published by Doghman et al. (GSE43035) [4] were retrieved using the GEOquery R package (v2.70.0) [15] [RRID:SCR_000146]. The gene expression matrix was available as log2 GC-RMA normalized expression values. Probe annotations were updated using the hugene10sttranscriptcluster.db R package (v8.8.0) [16].

When multiple probes mapped to the same gene, a single probe was retained based on the highest coefficient of variation across samples. Gene biotypes were subsequently filtered to retain protein-coding genes only.

Differential expression analysis was performed by comparing treatment and control groups defined in the original study using the limma R package (v3.58.1) [17] [RRID:SCR_010943].

The list of β-catenin knockdown DEGs in H295R cells was obtained from Lefèvre et al. [10]. Data for BAF inhibition in H295R and CU-ACC1 cells were retrieved from Chan et al. [13].

### 2.2. Pathway Enrichment Analysis of Perturbation Models

Pathway enrichment analyses were performed using curated gene sets from the Molecular Signatures Database (MsigDB) [18] [RRID:SCR_016863] and KEGG [19] [RRID:SCR_012773]. Hallmark and KEGG pathway collections were retrieved using the msigdbr (v26.1.0) [20] [RRID:SCR_022870] and KEGGREST (v1.48.1) [21] R packages, respectively.

Pathways related to steroidogenesis and WNT/β-catenin signaling were defined a priori based on keyword matching and manual curation, retaining the following gene sets: HALLMARK_WNT_BETA_CATENIN_SIGNALING, KEGG Steroid biosynthesis (hsa00100), KEGG Steroid hormone biosynthesis (hsa00140), KEGG Wnt signaling pathway (hsa04310) and KEGG Cortisol synthesis and secretion (hsa04927).

For each perturbation dataset (*NR5A1* knockdown, β-catenin knockdown and BAF inhibition), genes were ranked according to log2 fold change values derived from differential expression analyses. When necessary, gene identifiers were harmonized and filtered to retain protein-coding genes only.

Gene set enrichment analysis was performed using the fgsea R package (v1.34.2) [22] [RRID:SCR_020938]. Statistical significance was estimated using permutation-based testing.

### 2.3. TCGA-ACC Transcriptomic and Clinical Data

Transcriptomic and clinical data from patients with adrenocortical carcinoma (ACC) were obtained from The Cancer Genome Atlas (TCGA) cohort [6] [RRID:SCR_003193]. RNA-seq gene expression data were retrieved using the TCGAbiolinks R/Bioconductor package (v2.36.0) [23,24] [RRID:SCR_017683], which provides access to the Genomic Data Commons (GDC) repository [25] [RRID:SCR_014514]. Gene count matrices and CIMP classification were processed and organized using the SummarizedExperiment framework in R (v1.38.1) [26].

Overall survival (OS) data were obtained from the UCSC Xena Browser [27], which provides curated survival datasets for TCGA cohorts. Sample identifiers were harmonized between expression and clinical datasets using TCGA barcodes.

Gene expression matrices were filtered to retain protein-coding genes, based on annotations retrieved through the AnnotationHub R package (v3.16.1) [28] [RRID:SCR_024227]. Samples were then filtered and aligned with the 78 ACC samples for which regulatory activity information was available.

### 2.4. Transcriptional Regulatory Network

Regulon definitions used in this study were derived from the transcriptional regulatory network previously reconstructed for the TCGA-ACC cohort using the RTN framework (v2.35.1) [29,30], as described in our earlier work [31].

Briefly, the regulatory network was inferred using the ARACNe algorithm [32] [RRID:SCR_002180], which estimates transcription factor–target relationships based on mutual information between gene expression profiles. The network was constructed using a curated list of human transcription factors [33] and refined through permutation testing and bootstrap procedures implemented in the RTN pipeline.

Regulatory units (regulons) were defined as a transcription factor and its associated target genes. *NR5A1* and β-catenin related regulons (*LEF1*, *TCF7*, *TCF7L1* and *TCF7L2*) were selected for downstream analysis. Only regulons containing more than 15 positive and 15 negative targets were retained for downstream analyses [31].

### 2.5. Regulon Activity

Regulon activity was estimated using the two-tailed gene set enrichment analysis (GSEA2) method implemented in the RTN package within the Transcriptional Network Analysis (TNA) pipeline [29,30]. GSEA2 was applied both to the perturbation experiments (*NR5A1* knockdown, β-catenin knockdown and BAF inhibition in H295R and CU-ACC1 cells) and to estimate sample-specific regulon activity scores for each TCGA-ACC sample.

### 2.6. Evaluation of β-Catenin-Related Regulon Activity

To further validate the β-catenin-related regulons, their activities were compared between TCGA-ACC tumors with or without previously reported genomic alterations in the β-catenin pathway, including *CTNNB1*, *APC* and *ZNRF3* alterations [34], using the Wilcoxon rank-sum test. The regulons were also annotated against the HALLMARK_WNT_BETA_CATENIN_SIGNALING and KEGG Wnt signaling pathway gene sets using the tni.annotate.regulons() framework implemented in RTN (v2.35.1) [29]. Following the procedure implemented in the function, for each gene set, a sample-level expression score was calculated as the average expression of the genes from that set represented in the expression matrix. Samples were then divided into high- and low-gene-set-expression groups according to this score, using the median across the cohort as the cutoff. Pathway-associated gene expression signatures were then generated by comparing these two groups, and regulon enrichment was evaluated by two-tailed GSEA. Statistical significance was estimated using 10,000 permutations, followed by Benjamini–Hochberg correction.

### 2.7. Association Between Regulon Activity and CIMP Phenotype

Samples from the TCGA-ACC cohort were stratified into CIMP-high and CIMP low/intermediate groups, as previously described [6], based on the clinical annotations available for the TCGA-ACC dataset. Differences in *NR5A1* and β-catenin–related regulon activities between the two groups were assessed using the Wilcoxon rank-sum test [35,36].

To further evaluate the association between regulon activity and the CIMP-high phenotype, generalized linear models were fitted using a logistic regression framework, as implemented in the glm function in R [37,38]. We fitted separate logistic regression models testing *NR5A1* together with each β-catenin-related regulon. Specifically, each model included *NR5A1* regulon activity, one β-catenin-related regulon activity—*LEF1*, *TCF7* or *TCF7L2*—and the corresponding *NR5A1* × β-catenin-related regulon interaction term, using regulon activity scores (dES) as continuous predictors.

Model parameters were estimated assuming a binomial distribution with a logit link function and the statistical significance of individual coefficients was assessed using Wald tests. Interaction terms were included to evaluate whether the combined activity of two regulons was associated with the phenotype beyond their individual main effects.

### 2.8. Likelihood Ratio Test (LRT) for Interaction Between Regulons Activity

TCGA-ACC samples were classified as high or low activity for each *NR5A1* or β-catenin-related regulon according to the sign of their differential enrichment score (dES). Using the R package DESeq2 (v1.48.2) [39] [RRID:SCR_015687], for each *NR5A1*/β-catenin-related regulon pair, gene-level likelihood ratio tests were used to compare two nested models. The reduced model included the additive effects of *NR5A1* and the corresponding β-catenin-related regulon activity states, whereas the full model additionally included their interaction term. For each protein-coding gene, the likelihood ratio test assessed whether inclusion of the interaction term significantly improved model fit relative to the additive model. Genes with Benjamini–Hochberg-adjusted *p* values below 0.05 [40] were considered significant and were subsequently tested for enrichment against Hallmark and KEGG gene sets using the fgsea R package (v1.34.2) [22] [RRID:SCR_020938].

### 2.9. Survival and Interaction Analyses

Associations between regulon activity and clinical outcomes in 78 TCGA-ACC samples were evaluated using Cox proportional hazards models [41] implemented in the RTNsurvival R package (v1.35.1) [42]. Overall survival (OS) was used as the primary endpoint. To assess potential cooperative effects between transcriptional regulators, Cox models including multiplicative interaction terms between regulon activity scores were fitted.

Kaplan–Meier survival curves were generated using the RTNsurvival R package (v1.35.1) [42] to visualize overall survival according to combined regulon activity groups. Following the package’s default stratification procedure, each regulon was classified in each sample as having positive, negative, or undetermined activity according to the enrichment of its positively and negatively associated targets; activity was considered undetermined when both target sets showed enrichment scores with the same sign, precluding a consistent direction of regulon activity. For each regulon pair, samples were grouped as positive/positive, positive/negative, negative/positive, or negative/negative, whereas samples with undetermined activity for either regulon were assigned to a separate group. Differences between survival curves were assessed using the log-rank test [43,44].

### 2.10. Modulator Inference by Network Dynamics

We used the Modulator Inference by Network Dynamics (MINDy) algorithm implemented in the RTN R package [29] to evaluate whether curated transcriptional cofactors modulate the regulatory activity of *NR5A1* regulon [31]. A total of 389 curated cofactors, including *CTNNB1*, were initially considered as candidate modulators [45].

As previously described [29], MINDy assesses whether the mutual information between a transcription factor (TF) and its target genes changes as a function of the expression level of a candidate modulator. For each modulator, samples were stratified according to modulator expression, comparing the 35% of samples with the highest expression against the 35% with the lowest expression, using the default RTN parameters. Before statistical testing, candidate modulators were filtered using the default independence and range constraints implemented in RTN.

Statistical significance was assessed using the default MINDy framework, including Kolmogorov–Smirnov (KS) [46] and Fisher’s exact tests (FET) [47], with Bonferroni [48,49] correction for multiple testing. Results were ranked according to the adjusted Kolmogorov–Smirnov *p* value, as implemented by default in the RTN package [29].

### 2.11. Statistical Analyses

All analyses were performed in the R statistical environment (v4.5.1) [37] [RRID:SCR_001905] using packages available through CRAN and Bioconductor repositories [50] [RRID:SCR_006442]. Unless otherwise specified, multiple testing correction was conducted using the Benjamini–Hochberg false discovery rate (FDR) method [40] and adjusted *p* values below 0.05 were considered statistically significant.

## 3. Results

### 3.1. Enrichment of WNT/β-Catenin and Steroidogenic Pathways Under Perturbation

**Figure 1:**
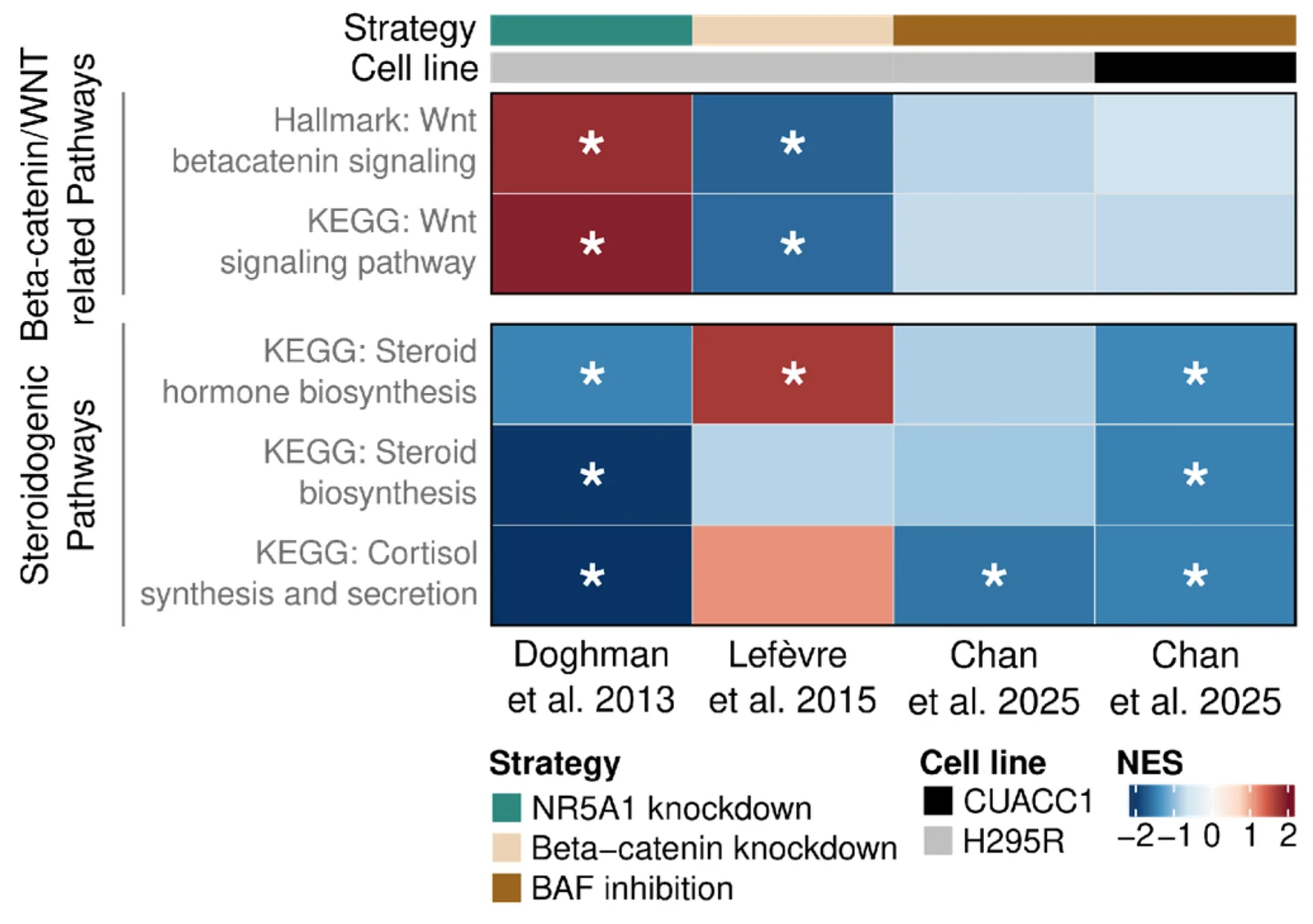
Enrichment of WNT/β-catenin and steroidogenic pathways across perturbation models. Heatmap showing normalized enrichment scores (NES) for selected Hallmark and KEGG gene sets following *NR5A1* knockdown in H295R cells [4], β-catenin knockdown in H295R cells [10] and BRM014-mediated BAF inhibition in H295R and CU-ACC1 cells [13]. Pathways were grouped as β-catenin/WNT-related or steroidogenic pathways. Colors indicate the direction and magnitude of enrichment, with negative NES values shown in teal and positive NES values shown in brown. Asterisks indicate significant enrichment after multiple-testing correction (adjusted *p* < 0.05). The top annotation indicates the perturbation strategy and the second annotation indicates the cell line used in each experiment.

We evaluated differential expression signatures derived from *NR5A1* and β-catenin knockdown experiments in H295R cells, as well as from BAF inhibition in H295R and CU-ACC1 cells, against steroidogenic and WNT/β-catenin–related pathways from the MSigDB Hallmark and KEGG collections (Figure 1). *NR5A1* knockdown resulted in significant negative enrichment across all steroidogenic pathways (KEGG Steroid hormone biosynthesis, KEGG Steroid biosynthesis and KEGG Cortisol synthesis and secretion), alongside significant positive enrichment of WNT/β-catenin signaling pathways (HALLMARK_WNT_BETA_CATENIN_SIGNALING and KEGG Wnt signaling pathway). In contrast, β-catenin knockdown showed suppression of WNT/β-catenin pathways, while displaying positive enrichment for KEGG Steroid hormone biosynthesis, with no significant association observed for the remaining steroidogenic pathways. BAF inhibition produced consistent negative enrichment across all evaluated pathways in both cell lines. Significant enrichment was observed for KEGG Cortisol synthesis and secretion in both H295R and CU-ACC1 cells, whereas KEGG Steroid hormone biosynthesis and KEGG Steroid biosynthesis reached significance only in CU-ACC1 cells.

### 3.2. NR5A1 and WNT/β-Catenin Related Regulons Activity Under Perturbation Experiments

**Figure 2:**
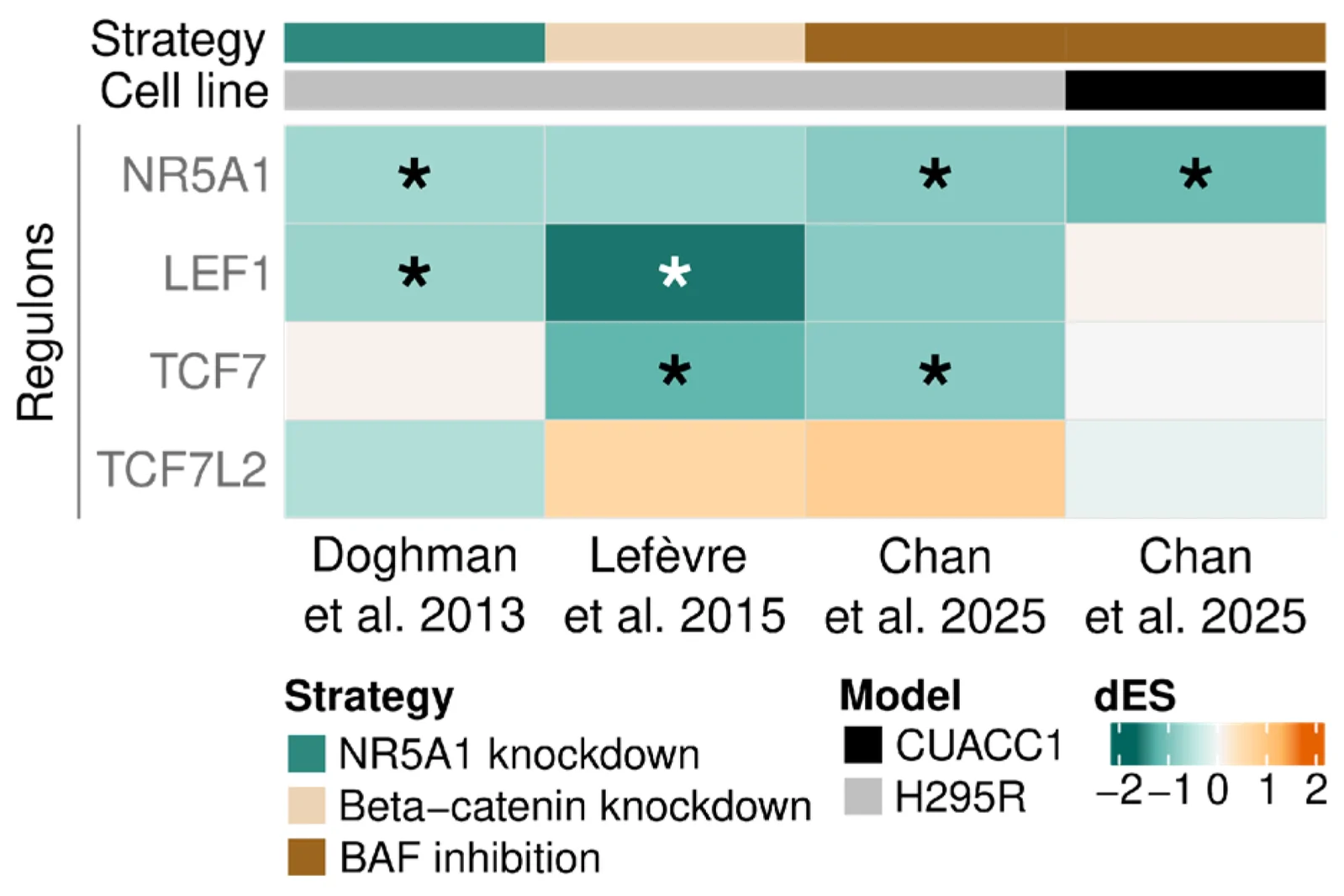
Activity of *NR5A1* and β-catenin-related regulons across perturbation models. Heatmap showing normalized enrichment scores (NES) for the *NR5A1*, *LEF1*, *TCF7* and *TCF7L2* regulons in *NR5A1* knockdown, β-catenin knockdown and BRM014-mediated BAF inhibition models. Regulon activity was inferred by two-tailed gene set enrichment analysis using regulons previously reconstructed from the TCGA-ACC cohort. Columns represent the perturbation datasets from Doghman et al. (2013) [4], Lefèvre et al. (2015) [10] and Chan et al. (2025) [13], with the top annotations indicating the perturbation strategy and cell line. Colors indicate the direction and magnitude of regulon activity, with negative NES values shown in purple and positive NES values shown in orange. Asterisks indicate significant enrichment after multiple-testing correction (adjusted *p* < 0.05).

We evaluated the activity of the *NR5A1* regulon and four β-catenin–related regulons (*LEF1*, *TCF7*, *TCF7L1* and *TCF7L2*) previously inferred from the TCGA-ACC cohort (Figure 2). Among these, *TCF7L1* did not meet the minimum requirement of 15 positive and 15 negative targets and was therefore excluded from subsequent analyses.

The *NR5A1* regulon showed negative enrichment across all tested conditions; however, statistical significance was not reached in the β-catenin knockdown (H295R). The *LEF1* regulon was significantly suppressed in the β-catenin knockdown, as expected. It was also suppressed in the *NR5A1* knockdown, while no significant enrichment was observed in the remaining conditions. *TCF7* was significantly suppressed in the β-catenin knockdown and in the BAF inhibition condition in H295R cells. Finally, *TCF7L2* did not show significant activity across the evaluated conditions.

### 3.3. β-Catenin-Related Regulon Activity and Association with Wnt/β-Catenin Pathways in TCGA-ACC

Of the 78 TCGA-ACC samples with regulon activity estimates, β-catenin pathway alteration status was available for 76 tumors; information was unavailable for TCGA-P6-A5OF and TCGA-PK-A5H8. *LEF1* and *TCF7L2* regulon activities were significantly higher in tumors harboring β-catenin pathway alterations, whereas no significant difference was observed for *TCF7*. In a complementary pathway-based analysis, all three regulons showed significant positive enrichment in transcriptional signatures associated with the HALLMARK_WNT_BETA_CATENIN_SIGNALING and KEGG Wnt signaling pathways (Supplementary Figures S1 and S2).

### 3.4. NR5A1 and WNT/β-Catenin Related Regulons Activity in TCGA-ACC CIMP Profiles

**Figure 3:**
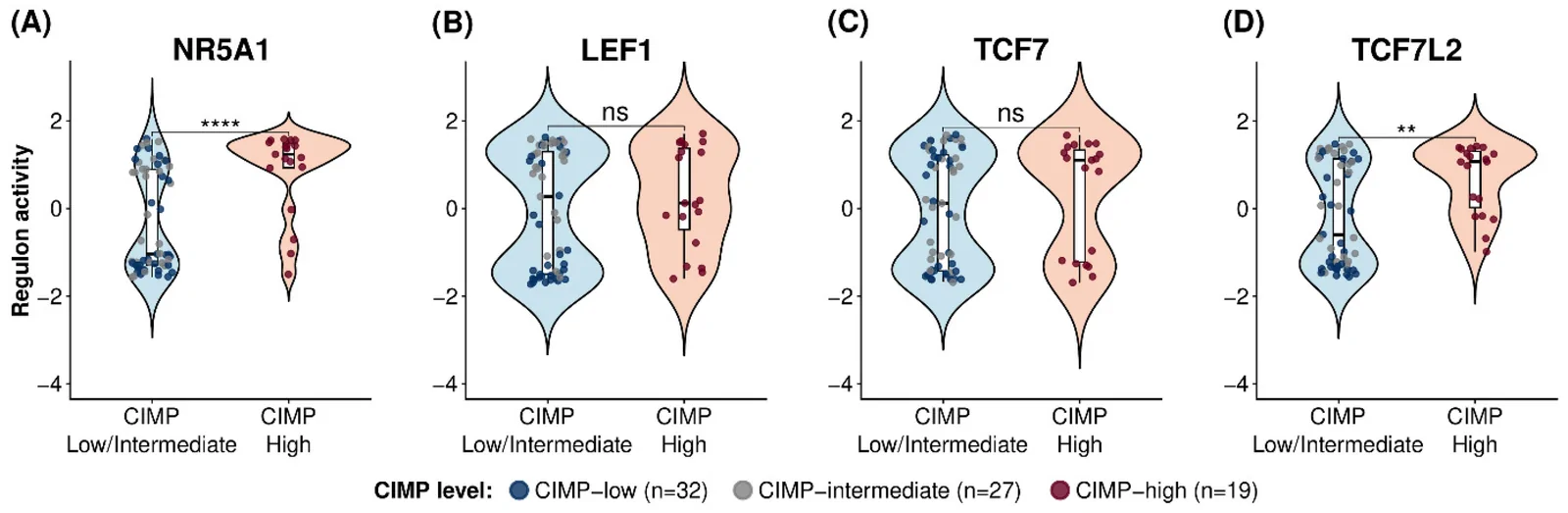
Distribution of *NR5A1* and β-catenin-related regulon activities according to CIMP status in TCGA-ACC. Violin plots show the distribution of regulon activity scores for (**A**) *NR5A1*, (**B**) *LEF1*, (**C**) *TCF7* and (**D**) *TCF7L2* in CIMP low/intermediate and CIMP-high tumors. Each point represents an individual sample and is colored according to CIMP subgroup. Group differences were assessed using the Wilcoxon rank-sum test. ** *p* < 0.01; **** *p* < 0.0001; ns, not significant.

Samples were stratified into CIMP low/intermediate and CIMP-high groups and regulon activity was compared between these profiles (Figure 3). Among the regulons evaluated, *NR5A1* and *TCF7L2* showed significantly higher activity in the CIMP-high group. In contrast, *TCF7* and *LEF1* did not exhibit significant differences between groups.

Logistic regression models evaluating the association between regulon activity and the CIMP-high phenotype consistently identified a significant main-effect coefficient for NR5A1 across all models that included *LEF1*, *TCF7* or *TCF7L2*. In contrast, neither the β-catenin-related regulons nor their interaction terms with *NR5A1* were significantly associated with the CIMP-high phenotype (Supplementary Table S1).

### 3.5. LRT for Interaction Terms

**Figure 4:**
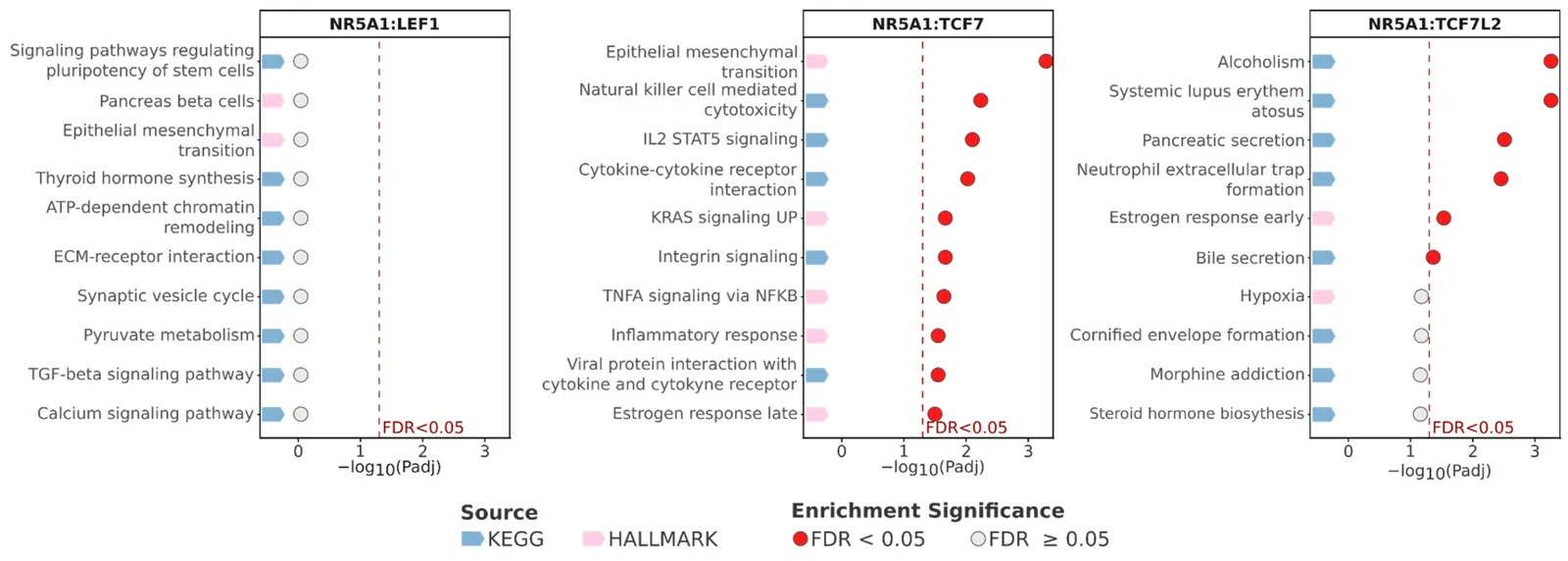
Pathway enrichment associated with *NR5A1*/β-catenin-related regulon interaction models. Pathway enrichment results for genes identified by likelihood ratio tests of the *NR5A1*:*LEF1*, *NR5A1*:*TCF7* and *NR5A1*:*TCF7L2* interaction models in TCGA-ACC. Each point represents a pathway, with farther-right positions indicating lower adjusted *p* values. Red points indicate significant enrichment (FDR < 0.05), whereas gray points indicate non-significant results. Colored markers beside pathway names denote the gene-set source (KEGG or Hallmark). The dashed line marks the FDR = 0.05 threshold.

To test whether the combined activity states of *NR5A1* and β-catenin-related regulons explained gene expression beyond their additive effects, we compared, for each gene in TCGA-ACC, models with and without an *NR5A1* × β-catenin-related regulon interaction term (Figure 4). Across all three comparisons, expression levels of fewer than 3% of the 24,233 protein-coding genes were significantly better explained by models including the interaction term than by additive models (Supplementary Table S2).

Gene sets derived from each LRT were subsequently evaluated for pathway enrichment using Hallmark and KEGG collections. No significant pathway enrichment was observed for the *NR5A1*:*LEF1* interaction. The *NR5A1*:*TCF7* interaction was associated with enrichment of immune-related pathways. In contrast, the *NR5A1*:*TCF7L2* interaction yielded six significantly enriched pathways, although no coherent biological pattern was identified (Figure 4). Notably, no steroidogenic or WNT/β-catenin–related pathways were significantly associated with any interaction term (Figure 4).

### 3.6. Interaction Between Regulons in Relation to Overall Survival

**Figure 5:**
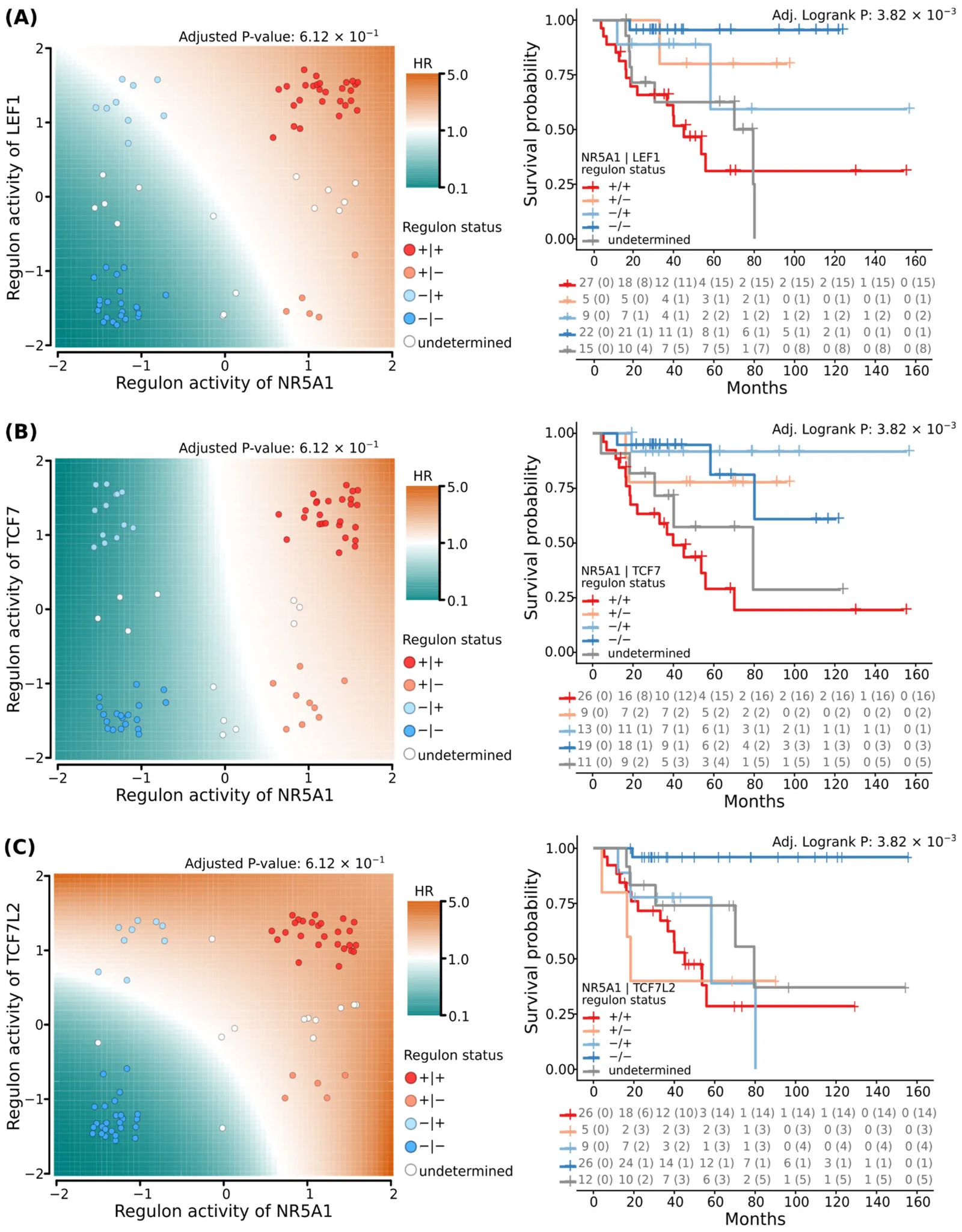
Overall survival according to combined *NR5A1* and β-catenin-related regulon activity in TCGA-ACC. Left panels show Cox proportional hazards model estimates across the continuous activities of *NR5A1* and (**A**) *LEF1*, (**B**) *TCF7* or (**C**) *TCF7L2* regulons. Points represent individual tumors classified according to combined high or low regulon activity. Adjusted *p* values for the corresponding multiplicative interaction terms are shown above each panel. Right panels show Kaplan–Meier overall survival curves for tumors stratified by combined high and low activity of *NR5A1* and the indicated β-catenin-related regulon. Log-rank *p* values are shown above the survival curves, and the numbers of patients at risk, with cumulative events shown in parentheses, are presented below each plot at the corresponding time points. Adjusted *p* values were obtained by Benjamini–Hochberg correction applied separately to the Cox interaction terms and to the log-rank tests, in each case across all pairwise regulon comparisons tested, including comparisons not displayed in this figure; identical adjusted values across panels are therefore expected. Raw p values are provided in Supplementary Tables S3 and S4.

In univariate Cox proportional hazards models, both *NR5A1* and *TCF7L2* regulon activities were significantly associated with overall survival, whereas no significant associations were observed for *TCF7* and *LEF1*. However, in multivariate Cox analyses, none of the interaction terms with *NR5A1* reached statistical significance (Figure 5, Supplementary Table S3).

Kaplan–Meier analyses stratifying samples according to combined *NR5A1* and β-catenin–related regulon activity showed that tumors with concurrent high activity of both regulons exhibited worse overall survival compared to other groups, whereas samples with low activity of both regulons exhibited improved overall survival (Figure 5). Complete statistical results for the Cox proportional hazards and Kaplan–Meier analyses are provided in Supplementary Tables S3 and S4 respectively.

### 3.7. Modulator Inference by Network Dynamics (MINDy)

**Figure 6:**
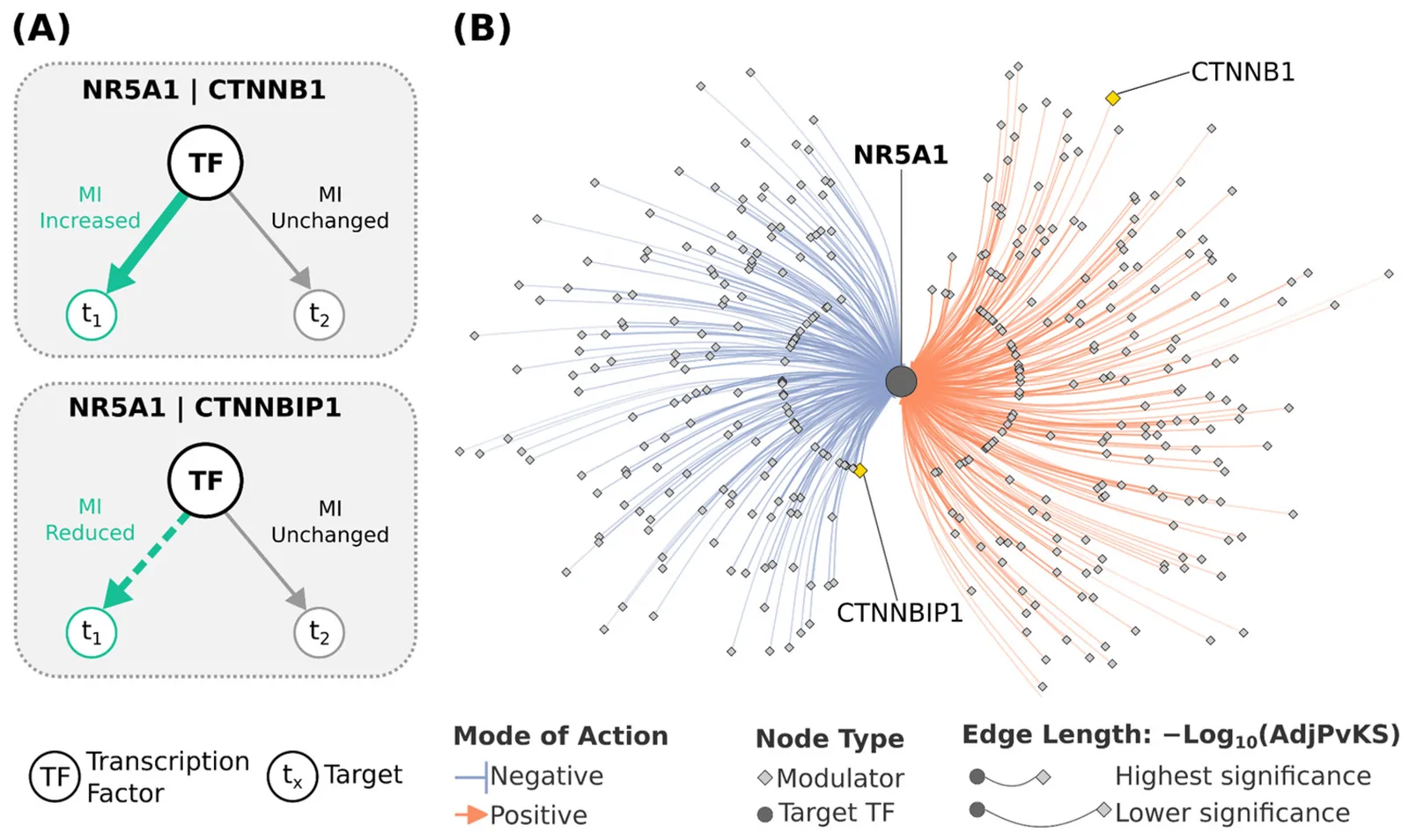
MINDy analysis of candidate modulators of *NR5A1* regulon activity in TCGA-ACC. (**A**) Schematic representation of the MINDy framework. Candidate modulators were assessed according to changes in mutual information (MI) between NR5A1 and its target genes across high- and low-modulator-expression groups. The notation NR5A1 | CTNNB1 or NR5A1 | CTNNBIP1 indicates that the mutual information between NR5A1 and its targets is evaluated conditionally on the expression of the indicated candidate modulator. CTNNB1 and CTNNBIP1 are illustrated as positive and negative modulators, respectively, while unchanged interactions are shown in gray. (**B**) Network representation of the curated cofactors evaluated as candidate modulators of the *NR5A1* regulon by MINDy analysis. *NR5A1* is shown as the central transcription factor and diamond-shaped nodes represent the tested cofactors. *CTNNB1* and *CTNNBIP1* are highlighted. Edge colors indicate the inferred mode of modulation of *NR5A1* regulon activity, with orange and blue edges representing positive and negative modulation, respectively. Edge length is proportional to the statistical significance of the MINDy result, expressed as −log10 of the adjusted *p* value. The complete list of tested cofactors and their MINDy results is provided in Supplementary Table S5.

We used the MINDy algorithm [29] to evaluate whether 389 curated cofactors modulate the regulatory activity of the *NR5A1* regulon in TCGA-ACC (Figure 6, Supplementary Table S5). Of these, six cofactors were not represented in the gene expression matrix (C14orf166, IKBKAP, MKL1, PARK2, TGFB1, and VPRBP), and 13 were excluded by the default independence/range constraints implemented in the MINDy analysis (HDAC9, SUMO1, WDR77, TRIM22, RPTOR, MED27, PRKCB, MALT1, PHF8, MAGEA2B, TLE1, LDB1, and MAGEA2). Among the 370 cofactors retained for statistical testing, *CTNNB1* ranked 355th among the tested cofactors and was associated with significant positive modulation of *NR5A1* regulon activity (adjusted FET *p* value = 2.87 × 10^−5^; adjusted KS *p* value = 3.66 × 10^−5^), despite its low relative ranking. *CTNNB1* expression was associated with modulation of 46 positive and 25 negative NR5A1 targets.

In addition to *CTNNB1*, the primary β-catenin-related candidate evaluated, *CTNNBIP1* was identified among the top 10% of *NR5A1* modulators. *CTNNBIP1* was associated with significant negative modulation of the *NR5A1* regulon (adjusted FET *p* value = 1.09 × 10^−34^; adjusted KS *p* value < 1 × 10^−14^), affecting 136 *NR5A1* targets, with a predominantly negative pattern comprising 118 negative and 18 positive effects. The complete ranked MINDy results for the *NR5A1* regulon are presented in Supplementary Table S5.

## 4. Discussion

The functional interaction between *CTNNB1* and *NR5A1* has been proposed to involve regulatory mechanisms operating at transcriptional, epigenetic and signaling-network levels. In this computational analysis study, we evaluated transcriptional output using regulatory network inference, perturbation-based regulon activity, CIMP association, survival analyses, gene-level interaction models and modulator inference.

Transcriptomic signatures from *NR5A1* and β-catenin knockdown models [4,10] were analyzed against their respective target pathways. Given the established role of *NR5A1* in adrenal steroidogenesis and the canonical association of β-catenin with WNT signaling, steroidogenesis- and WNT/β-catenin-related pathways were selected as an initial framework to evaluate whether β-catenin perturbation would modulate established *NR5A1*-associated functions. *NR5A1* knockdown significantly suppressed steroidogenic gene sets, while β-catenin knockdown selectively repressed canonical WNT/β-catenin signaling. Importantly, neither perturbation model resulted in the cross-repression of the other’s primary regulatory axis.

Concerning the regulons activity in the perturbation models, the *NR5A1* regulon was significantly repressed after *NR5A1* knockdown, while β-catenin knockdown was concordant with repression of *LEF1* and *TCF7* regulon activity, but did not show a clear directional enrichment for the *TCF7L2* regulon. Although β-catenin knockdown showed negative enrichment of *NR5A1* regulon activity, this effect did not reach statistical significance. Overall, these perturbation-based analyses did not provide evidence that *NR5A1* and β-catenin knockdown responses converge on a shared transcriptional output within the steroidogenesis- and WNT/β-catenin-related canonical pathways that were evaluated in ACC cells.

In the TCGA-ACC analyses, TCF/LEF regulon activities were used as indirect measures of canonical β-catenin-related transcriptional output rather than as direct measurements of β-catenin activation. The higher activities of the LEF1 and TCF7L2 regulons in tumors harboring Wnt/β-catenin pathway alterations, together with the positive enrichment of all three regulons in established Wnt-related gene signatures, provide complementary support for their use as proxies of canonical pathway activity. Nevertheless, these regulons capture only the transcriptional component mediated through TCF/LEF factors and should not be interpreted as comprehensive measures of all β-catenin functions.

Regarding CIMP profiles, *NR5A1* regulon activity was consistently associated with the CIMP-high phenotype across both univariate and multivariate models, whereas β-catenin–related regulon interaction coefficients did not reach statistical significance after adjustment for NR5A1 activity. Although *TCF7L2* activity was increased in CIMP-high tumors in Wilcoxon analysis, this association was not retained after adjustment for *NR5A1* in logistic models and no interaction terms reached statistical significance. These findings indicate that the transcriptional signal associated with CIMP-high is predominantly captured by *NR5A1* activity, with no evidence of coordinated contribution from TCF/LEF–related regulons.

Kaplan–Meier analyses showed that tumors with concurrent activation of *NR5A1* and β-catenin–related regulons were associated with worse overall survival. However, within the multivariate Cox proportional hazards analysis, none of their interaction terms with *NR5A1* reached statistical significance. This indicates that the combinatorial effect observed in univariate stratification does not retain statistical significance after adjusting for the *NR5A1* regulon activity.

To examine broader transcriptional contexts beyond pairwise regulon interaction models, we applied the conditional mutual information-based MINDy algorithm to identify candidate modulators of *NR5A1* regulatory activity. *CTNNB1*, encoding β-catenin, was identified as a statistically significant positive modulator of the *NR5A1* regulon. However, among the 370 curated cofactors analyzed, *CTNNB1* ranked 355th, indicating that *CTNNB1* expression alone does not emerge as a dominant modulator of *NR5A1* regulatory activity within this computational framework. In contrast, *CTNNBIP1* was identified among the top 10% of *NR5A1* modulators and was associated with a significant negative modulation of the *NR5A1* regulon. *CTNNBIP1*/ICAT is a β-catenin-interacting protein known to inhibit β-catenin-mediated transcription by disrupting β-catenin-containing transcriptional complexes [51]. Therefore, this finding points to a transcriptomic association between a β-catenin-inhibitory regulatory context and reduced *NR5A1* activity, although its mechanistic relevance requires further investigation.

In addition to *CTNNBIP1*-associated negative modulation, we observed a significant repression of the *NR5A1* regulon upon cBAF complex inhibition via BRM014 in both H295R and CU-ACC1 cell lines. This observation is consistent with results presented by Chan et al. [13]. Nevertheless, while cBAF inhibition represses *NR5A1* regulon activity, the precise dependency of *NR5A1*/cBAF chromatin interactions on β-catenin remains unresolved. Given the limited global transcriptomic convergence between *NR5A1*- and β-catenin-regulated genes, whether β-catenin acts as an obligatory structural bridge at these loci, or whether β-catenin-independent recruitment mechanisms involving alternative cofactors also contribute, remains a mechanistic question requiring further empirical validation.

It is important to remark that non-canonical Wnt signaling pathways, which operate independently of β-catenin transcriptional activation, may indirectly influence *NR5A1* activity through modulation of intracellular calcium signaling, planar cell polarity pathways, cytoskeletal organization, cellular metabolism or kinase-mediated signaling cascades such as MAPK, PI3K/AKT and JNK [52,53,54]. Conversely, *NR5A1* itself could modulate responsiveness to both canonical and non-canonical Wnt signaling by maintaining adrenal lineage identity and regulating expression of pathway components or cofactors [3,55]. These interactions are likely highly context dependent, varying according to developmental stage, steroidogenic phenotype, mutational background, chromatin architecture and tumor subtype, which may explain the heterogeneous observations reported across experimental systems and computational analyses [5,6,13]. Because our analyses focused on canonical β-catenin/TCF-LEF transcriptional output, they do not address β-catenin-independent Wnt signaling or other signaling pathways that could indirectly influence *NR5A1* activity. These possibilities remain outside the scope of the present study.

Several limitations of our work should be acknowledged. First, regulon activity inference captures downstream transcriptional output and may not reflect protein–protein interactions, post-translational regulation, chromatin accessibility or enhancer occupancy. Second, in the interaction analysis, β-catenin activity was approximated primarily through TCF/LEF-associated regulons, which represent canonical WNT/β-catenin transcriptional output but may not capture non-canonical β-catenin functions. While TCF/LEF factors have been shown to mediate the largest proportion of β-catenin transcriptional output [8], non-canonical β-catenin functions, including its roles in adherens junctions, cytoskeletal organization and context-specific co-activator interactions, are not captured by this proxy. Third, the TCGA-ACC cohort is relatively small (*n* ≈ 78), which limits statistical power to detect modest interaction effects, particularly in survival analyses and likelihood ratio tests. It is therefore possible that weak interaction signals between *NR5A1* and β-catenin–related regulons fall below the detection threshold of the present analyses. Fourth, the perturbation experiments used to evaluate pathway concordance (*NR5A1* knockdown, β-catenin knockdown, BRM014 treatment) were conducted in established cell line models (H295R, CU-ACC1), which may not fully recapitulate the transcriptional landscape of primary tumors. Extrapolation of these in vitro findings to the TCGA cohort should therefore be made with caution. Finally, the use of overall survival as the primary clinical endpoint is subject to confounding by treatment heterogeneity across the TCGA cohort and the relatively limited follow-up available in this dataset may reduce the sensitivity of survival analyses.

In summary, this study provides a multi-layered evaluation of the transcriptional relationship between *NR5A1* and β-catenin in ACC. Our results indicate that *NR5A1* regulon activity was associated with CIMP-high status and overall survival, whereas statistically significant interaction effects involving the β-catenin-related regulons were not detected in the bulk TCGA-ACC analyses. Given the limited cohort size, these findings do not exclude more modest or context-dependent effects. MINDy analysis adds a further layer by showing that *CTNNB1* expression is a statistically significant but low-ranking positive modulator of *NR5A1*, whereas *CTNNBIP1*, an inhibitor of β-catenin-mediated transcription, is a negative modulator of the *NR5A1* regulon. These findings suggest that the relationship between *NR5A1* and β-catenin is not captured by canonical TCF/LEF regulon activity or *CTNNB1* transcript abundance alone. These results provide a cautious basis for interpreting the absence of significant transcriptional interaction signals in bulk data with prior mechanistic studies proposing more localized, context-dependent regulatory interactions, thereby establishing a rationale for future biochemical and genomic studies to test whether such additional regulatory layers contribute to *NR5A1*/β-catenin relationships in ACC.

## Data Availability

Publicly available datasets were analyzed in this study. TCGA-ACC RNA-seq and clinical data are available through the Genomic Data Commons and UCSC Xena. The *NR5A1* knockdown dataset is available from the Gene Expression Omnibus [RRID:SCR_005012] under accession number GSE43035. Differential expression data for β-catenin knockdown and BRM014-mediated cBAF inhibition were obtained from the Supplementary Materials of [10] and [13], respectively. The complete MINDy results are provided in Supplementary Table S5. The R scripts used for the analyses are available from the corresponding author upon reasonable request.

## Supplementary Materials

The following supporting information can be downloaded at: https://www.mdpi.com/article/10.3390/medsci14040493/s1: Figure S1: β-catenin-related regulon activity according to β-catenin pathway alteration status in TCGA-ACC. Violin plots show the distribution of regulon activity scores for (A) LEF1, (B) TCF7, and (C) TCF7L2 in tumors with genomic alterations affecting the β-catenin pathway (*n* = 41) and in tumors without such mutations (*n* = 35). Individual points represent tumor samples, and the internal boxplots indicate the median and interquartile range. Group differences were assessed using the Wilcoxon rank-sum test. ** *p* < 0.01; *** *p* < 0.001; ns, not significant. Figure S2: Enrichment of β-catenin-related regulons in canonical Wnt/β-catenin gene-expression signatures in TCGA-ACC. Two-tailed gene set enrichment analysis was used to evaluate the LEF1, TCF7, and TCF7L2 regulons against transcriptional signatures associated with the HALLMARK_WNT_BETA_CATENIN_SIGNALING gene set (A–C, respectively) and the KEGG Wnt signaling pathway gene set (D–F, respectively). In each panel, genes are ranked according to the corresponding pathway-associated expression phenotype. Positively and negatively associated regulon targets are indicated by red and blue marks, respectively, along the ranked gene list, and the corresponding enrichment profiles are shown below. Differential enrichment scores (dES) and adjusted *p* values are displayed within each panel. All three regulons showed significant positive enrichment in both Wnt/β-catenin-related transcriptional signatures. Table S1: Logistic regression analyses of the association between regulon activity and the CIMP-high phenotype in TCGA-ACC. The table includes separate models combining NR5A1 with the LEF1, TCF7 or TCF7L2 regulons. For each model, odds ratios, 95% confidence intervals and *p* values are provided for the NR5A1 regulon, the corresponding β-catenin-related regulon and their multiplicative interaction term. A summary of the interaction terms and their Benjamini–Hochberg-adjusted *p* values is also included. Table S2: Gene-level likelihood ratio test results for interactions between NR5A1 and β-catenin-related regulon activity in TCGA-ACC. The table includes results for the NR5A1 × LEF1, NR5A1 × TCF7 and NR5A1 × TCF7L2 interaction models. For each protein-coding gene, the Ensembl gene identifier, gene symbol, mean normalized expression, likelihood ratio statistic, *p* value and Benjamini–Hochberg-adjusted *p* value are provided. Table S3: Cox proportional hazards analyses of regulon activity in TCGA-ACC. The table includes univariate Cox models for NR5A1, LEF1, TCF7 and TCF7L2 regulon activities, as well as multivariate models combining NR5A1 with each β-catenin-related regulon and the corresponding multiplicative interaction term. Hazard ratios, 95% confidence intervals and *p* values are provided. Summaries of the interaction terms and their Benjamini–Hochberg-adjusted *p* values are also included. Table S4: Numbers at risk and cumulative events for Kaplan–Meier analyses of combined regulon activity groups in TCGA-ACC. The table includes results for all pairwise combinations of the NR5A1, LEF1, TCF7 and TCF7L2 regulons. For each regulon pair, samples were classified into positive/positive, positive/negative, negative/positive, negative/negative or undetermined activity groups according to the default RTNsurvival procedure. Values indicate the number of samples at risk, followed in parentheses by the cumulative number of events, at 20-month intervals from 0 to 140 months. Global log-rank statistics, *p* values and Benjamini–Hochberg-adjusted *p* values are also provided. Table S5: Complete MINDy results for candidate modulators of the NR5A1 regulon in TCGA-ACC. The table includes all curated cofactors evaluated by MINDy, their rank based on the adjusted Kolmogorov–Smirnov *p* value, the adjusted Kolmogorov–Smirnov and Fisher’s exact test *p* values, the inferred mode of modulation and the numbers of positively and negatively modulated NR5A1 regulon targets.

## Abbreviations

The following abbreviations are used in this manuscript:

ACC	Adrenocortical Carcinoma
CIMP	CpG Island Methylator Phenotype
DEG	Differentially Expressed Gene
GSEA	Gene Set Enrichment Analysis
LRT	Likelihood Ratio Test
MINDy	Modulator Inference by Network Dynamics
TCGA	The Cancer Genome Atlas
GEO	Gene Expression Omnibus
MSigDB	Molecular Signatures Database
KEGG	Kyoto Encyclopedia of Genes and Genomes
RTN	Reconstruction of Transcriptional Networks
TNA	Transcriptional Network Analysis
ARACNe	Algorithm for the Reconstruction of Accurate Cellular Networks
NES	Normalized Enrichment Score
dES	Differential Enrichment Score
FDR	False Discovery Rate
cBAF	Canonical BRG1/BRM-Associated Factor
KS	Kolmogorov–Smirnov
FET	Fisher’s Exact Test
OS	Overall Survival
CRAN	Comprehensive R Archive Network
GDC	Genomic Data Commons
