# Supplementary material for: Distinct Transcriptional Programs Controlled by NR5A1 and β-Catenin in Adrenocortical Carcinoma
Source: Med Sci (Basel). 2026 Aug 19;14(4):493. doi: 10.3390/medsci14040493 (PMC13515156; doi:10.3390/medsci14040493)
Supplement: Supplementary file 1 [file medsci-14-00493-s001.zip › Supplementary Figures.pdf]

medical sciences

MDPI

Supplementary Figures

Distinct Transcriptional Programs Controlled by *NR5A1* and β-Catenin in Adrenocortical Carcinoma

João Carlos Degraf Muzzi[1,2,*], Bonald Cavalcante Figueiredo[1,3], Jean Silva de Souza Resende[1,2,3], Igor Samesima Giner[1,2,3], Mauro Antônio Alves Castro[2,*], Enzo Lalli[4,*]

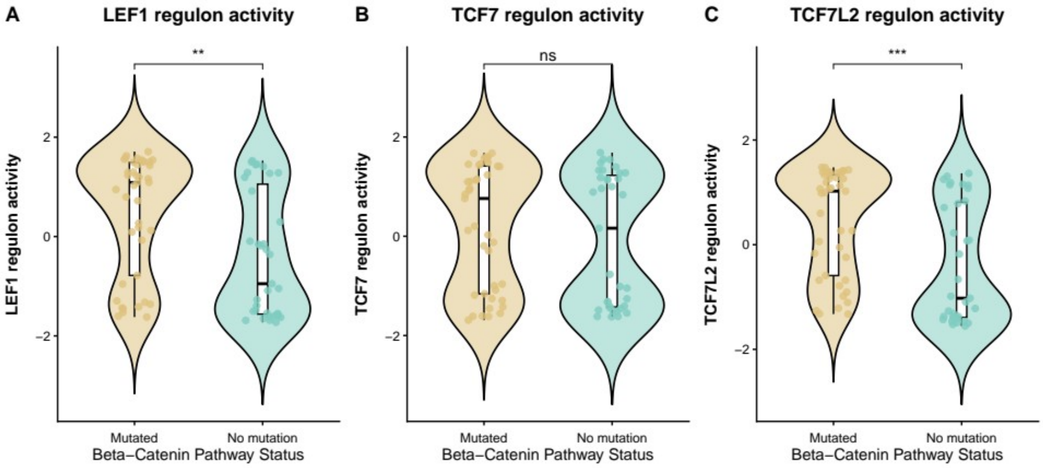

Supplementary Figure S1. β-catenin-related regulon activity according to β-catenin pathway alteration status in TCGA-ACC. Violin plots show the distribution of regulon activity scores for (A) LEF1, (B) TCF7, and (C) TCF7L2 in tumors with genomic alterations affecting the β-catenin pathway (n=41) and in tumors without such mutations (n=35). Individual points represent tumor samples, and the internal boxplots indicate the median and interquartile range. Group differences were assessed using the Wilcoxon rank-sum test. **P < 0.01; ***P < 0.001; ns, not significant.

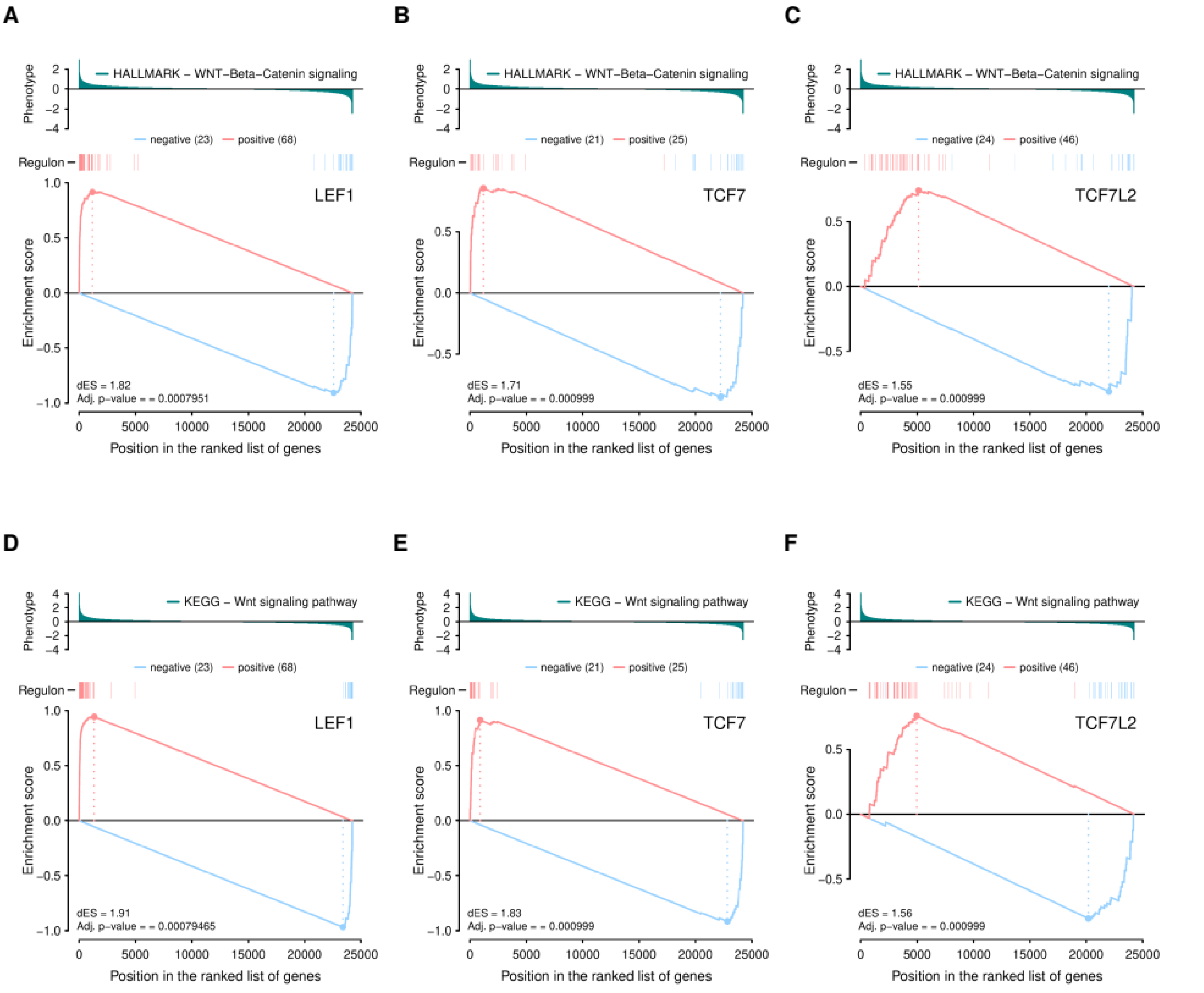

Supplementary Figure S2. Enrichment of β-catenin-related regulons in canonical Wnt/β-catenin gene-expression signatures in TCGA-ACC. Two-tailed gene set enrichment analysis was used to evaluate the LEF1, TCF7, and TCF7L2 regulons against transcriptional signatures associated with the HALLMARK_WNT_BETA_CATENIN_SIGNALING gene set (A–C, respectively) and the KEGG Wnt signaling pathway gene set (D–F, respectively). In each panel, genes are ranked according to the corresponding pathway-associated expression phenotype. Positively and negatively associated regulon targets are indicated by red and blue marks, respectively, along the ranked gene list, and the corresponding enrichment profiles are shown below. Differential enrichment scores (dES) and adjusted P values are displayed within each panel. All three regulons showed significant positive enrichment in both Wnt/β-catenin-related transcriptional signatures.
